# P05.27. Older peoples’ use of complementary and alternative medicine to cope and ‘keep going’

**DOI:** 10.1186/1472-6882-12-S1-P387

**Published:** 2012-06-12

**Authors:** N Robinson, A Lorenc

**Affiliations:** 1 London South Bank University, London, United Kingdom

## Purpose

To explore older peoples’ decision-making regarding their experiences of complementary and alternative medicine (CAM).

## Methods

This paper uses data from two separate community groups. Focus groups with volunteers aged over 58 explored perceptions of health and wellbeing. They were carried out in two community centers in southwest (3 focus groups) and northeast London (1 focus group). The first three groups specifically targeted healthcare decision making, and the fourth covered the experiences of participants in a taiji class. Data was content analyzed using Framework analysis and Atlas ti.

## Results

Thirty-seven people participated in the three decision-making focus groups, 8 in the taiji group. Regarding decision-making, five themes emerged; physical wellbeing, impact on activity, emotional issues, community and health services, and keeping positive. A range of CAM was used, commonly mind/body or physical therapies. The main reason was to ‘keep going’ and maintain wellbeing. Conventional medicine was perceived as central to wellbeing, with CAM used to address its limitations. Decision making was rarely systematic; anecdotal information dominated, and disclosure to conventional practitioners was uncommon. Results from the taiji focus group reinforced these themes and the importance of CAM (taiji) as a ‘tool’ for coping with day-to-day problems, perceived as helpful for mobility, sleep and relaxation.

## Conclusion

‘Keeping going’ is important for older people and often promoted by CAM, including manipulative and exercise therapies such as taiji. Concurrent CAM and conventional medication use, unreliable information and insufficient discussion with conventional providers may have safety implications. Older people perceive great benefit from CAM such as taiji, and often use it as a self-management tool. Healthcare practitioners should consider exploring CAM use with older people and facilitating access to CAM information. Asking older people about CAM use may be integral to providing holistic, safe care.

